# A Japanese Nationwide Survey of Nutritional Counseling for Cancer Patients and Risk Factors of Burnout among Registered Dietitians

**DOI:** 10.1089/pmr.2022.0038

**Published:** 2022-09-27

**Authors:** Haruka Chitose, Miki Kuwana, Tomofumi Miura, Mihoko Inoue, Yuko Nagasu, Ryogo Shimizu, Yukako Hattori, Yuko Uehara, Kazuhiro Kosugi, Yoshihisa Matsumoto

**Affiliations:** ^1^ Nutrition Management Office, National Cancer Center Hospital East, Kashiwa, Japan.; ^2^ Department of Palliative Medicine, National Cancer Center Hospital East, Kashiwa, Japan.; ^3^ Nutrition Management Office, Tochigi Medical Cancer, Utsunomiya, Japan.; ^4^ Department of Hematology/Oncology, Yamato Municipal Hospital, Yamato, Japan.; ^5^ Department of Palliative Medicine, Juntendo University Graduate School of Medicine, Tokyo, Japan.; ^6^ Cancer Therapeutic Center, Juntendo University Urayasu Hospital, Chiba, Japan.

**Keywords:** burnout, cancer, dietitian, nutritional counseling, palliative care

## Abstract

**Purpose:**

Registered dietitians have played a key role in the nutritional management of cancer patients; however, no study has investigated the prevalence of burnout and associated factors among this population. The aim of this study was to investigate the following: (1) experiences, approaches, and perspectives during nutritional counseling, (2) the prevalence of burnout, and (3) burnout-associated factors among registered dietitians.

**Methods:**

A nationwide survey with self-administered questionnaires was conducted for 1070 registered dietitians belonging to all 390 designated cancer hospitals in Japan. Nutrition counseling, the prevalence of burnout, and burnout-associated factors were analyzed.

**Results:**

A total of 631 responses were analyzed. Half of the respondents recommended a consultation about treatment of symptoms or listened to their patients' distress and anxiety of death. Respondents with a severe level of burnout for emotional exhaustion, depersonalization, and personal accomplishment (PA) were 21.1%, 2.8%, and 71.9%, respectively. Burnout was associated with fewer years of clinical experience, more overtime hours, higher score of Patient Health Questionnaire-9, higher score of K-6, not so positive attitude about caring for dying patients, the approach of listening to the patients and family distress and anxiety about death, the uneasiness interacting with patients and families without an effective proposal, the difficulty of allocating staff without increasing medical costs, and the absence of feeling of a good contribution to patients and families.

**Conclusions:**

The prevalence of burnout on PA was quite high. Registered dietitians who engaged in nutritional counseling for cancer patients and families might benefit from education to help protect against burnout.

## Introduction

Cancer is the leading cause of death in the world. Malnutrition is frequently seen in cancer patients (20%–70%) depending on factors such as their diagnosis, stage, age, and method of assessment.^1^ Malnutrition has negative impacts on cancer patients with poor prognosis,^2^ resistance to antitumor treatment,^3^ increased therapy toxicity,^4^ and impaired physical function^5^ and quality of life (QOL).^5^ The present guidelines recommend nutritional screening to detect a risk of malnutrition.^1^ Nutritional assessment and nutritional intervention are recommended for patients with a risk of malnutrition. A previous meta-analysis reported that nutritional intervention can improve global QOL, loss of appetite, and emotional function.^6^ Nutritional interventions include nutritional counseling, oral nutritional supplements, artificial nutrition, symptom management, and drug therapy.

The first strategy for cancer patients with a risk of malnutrition is nutritional counseling.^1^ In the present health care system, registered dietitians are considered a key member of the nutrition support team and play a central role in nutritional management including nutritional counseling. Through nutritional management, registered dietitians are involved with cancer patients through their illness trajectory. However, registered dietitians working among cancer patients and their families experience eating-related distress; thus, they may be working in similar high-stress environments to other health care professionals in the oncology and palliative care fields.^7–9^

Burnout is a psychological syndrome of emotional exhaustion (EE), depersonalization (DP), and reduced personal accomplishment (PA).^10^ Burnout has negative impacts on the well-being and QOL of health care professionals, and increases the risk of job withdrawal, absenteeism, and suicidal ideation.^11–14^ Previous studies also reported that burnout decreased quality of care and increased medical errors.^11,15,16^ Among oncology staff, burnout is well known and has been reported in 38% to 56% of oncologists,^11,17,18^ 20% of nurses,^11^ and 16% of medical social workers.^11^ A previous study reported stress and burnout among registered dietitians^19^; however, no study has shown the prevalence of burnout and associated factors among registered dietitians.

The aim of this study was to reveal the following: (1) experiences, approaches, and perspectives during nutritional counseling, (2) the prevalence of burnout, and (3) burnout-associated factors among registered dietitians.

## Methods

This study was a nationwide cross-sectional survey with self-administered questionnaires for registered dietitians conducted between September and November 2020. This study was conducted according to the Declaration of Helsinki and was approved by the Institutional Review Board of the National Cancer Center, Japan (approval no. 2020-009).

### Procedures

Envelopes including three questionnaires were mailed to the chief dietitian at all of the 390 designated cancer hospitals in Japan. At each hospital, the chief dietitian invited other two additional registered dietitians including one moderately experienced registered dietitian, and one inexperienced registered dietitian who had been involved in nutritional counseling for cancer patients. Therefore, a total of three registered dietitians were asked to separately complete the questionnaires and individually return them using each envelope until November 2020.

### Measurements

#### Sociodemographic and clinical characteristics

The questionnaire was developed through discussions within our research organization. It included sex, years of clinical experience, number of beds, number of registered dietitians, and overtime hours per month.

#### Psychological questionnaires

The participants' burnout was assessed using the Maslach Burnout Inventory Human Services Survey (MBI-HSS).^20^ MBI-HSS is a 22-item, 7-point Likert scale. MBI-HSS has three subscales of burnout as follows: EE, DP, and PA, following the scoring manual. Higher scores of EE and DP and lower scores of PA are related with higher levels of burnout. The participants' psychological distress was evaluated using the Japanese version of the K6 scale.^21^ The K6 is a 6-item, 5-point Likert scale. The total K6 score ranges from 0 to 24, with higher scores indicating more severe psychological distress, and serious mental distress defined as a score of 13 or more. The participants' depression was evaluated using the Patient Health Questionnaire-9 (PHQ-9). The PHQ-9 is a 9-item, 4-point Likert scale.^22^

The Japanese version of the PHQ-9 has been validated.^23^ A PHQ-9 score of 10 or more is defined as depression. The attitudes to caring for the dying patients were evaluated using the short version of the Frommelt Attitude Toward Care of the Dying - Form B—Japanese (FATCOD-B-J).^24^ The short version of FATCOD-B-J is a 6-item, 5-point Likert scale. The short version of FATCOD-B-J has two independent dimensions: positive attitude toward caring for the dying patient (FATCOD I) and perception of patient- and family-centered care (FATCOD II).

#### Experiences, approaches, and perspectives during nutritional counseling

The questionnaire using a 6-point Likert scale (1, strongly disagree to 6, strongly agree) was originally developed through discussions among researchers to obtain information on the patient's experiences, approaches, perspectives, and hopes during nutritional counseling. The experiences included: “Patient says ‘I want to eat but I can't’,” “Patient says ‘I'm full as soon as I eat’,” and “Patient says ‘I feel nausea as soon as I eat’.” The approaches included: “I propose specific recipes for patients and families,” “I introduce cooking classes for patients and families,” and “I propose dietary nutritional supplements.” The perspectives included: “I think I can contribute to patients and families,” “I empathize with the eating-related distress of patients and families,” and “I want to get rid of the eating-related distress of patients and families.”

### Statistical analysis

Descriptive analysis was performed to assess the background of participants and the results of the questionnaires. For each item about experiences, approaches, and perspectives in the questionnaire, responses were dichotomized into a disagree group (1, strongly disagree to 3, slightly disagree) and an agree group (4, slightly agree to 6, strongly agree), respectively. To explore the potential factors for burnout among registered dietitians, univariate analyses for EE, DP, and PA were performed using analysis of variance or chi-square test. Furthermore, to identify independent factors for burnout among registered dietitians, all factors with *p* < 0.1 identified in univariate analyses were used in multivariate linear regression analyses for EE, DP, and PA. Significance was set as *p* < 0.05. All analyses were performed using JMP 14.2 (SAS Institute, Cary, NC).

## Results

### Respondent background

**FIG. 1.**
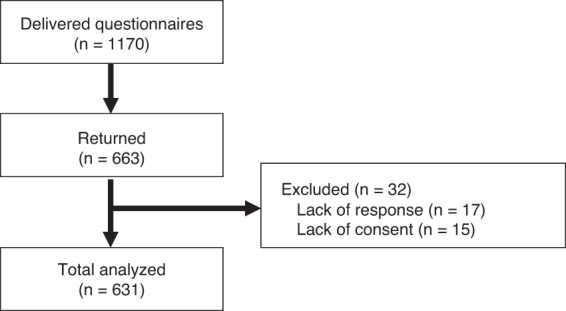
Participant flow according to STROBE statements.

A total of 663 questionnaires were returned (response rate; 56.7%). We excluded 17 participants who did not respond and 15 participants without consent; therefore, 631 participants were analyzed (Fig. 1). Participants' background is shown in Table 1. The mean years of clinical experience was 15.9 years (standard deviation [SD] = 10.0) and 546 (89.8%) were female. The most common number of beds was 401 to 600 beds (39.3%). The most common number of registered dietitians was 7 to 10 (41.7%). The mean overtime hours per month was 15.5 hours (SD = 14.9).

**Table 1.** Background of Participants

	***N***	(%)
Sex		
Male	62	(10.2)
Female	546	(89.8)
Years of clinical experience		
Mean (SD)	15.9	(9.9)
No. of beds		
≤200	8	(1.3)
201–400	128	(21.1)
401–600	248	(40.9)
601–1000	193	(31.8)
>1000	29	(4.8)
No. of registered dietitians		
≤3	24	(4.0)
4–6	169	(27.8)
7–10	263	(43.3)
>10	151	(24.9)
Overtime hours per month		
Mean (SD)	15.5	(14.9)
PHQ-9 score		
Mean (SD)	5.6	(4.4)
<10	513	(82.1)
≥10	112	(17.9)
K-6 score		
Mean (SD)	3.7	(4.0)
<13	580	(95.9)
≥13	25	(4.1)
MBI-HSS emotional exhaustion		
Mean (SD)	19.7	(11.2)
Low	299	(48.6)
Moderate	186	(30.2)
High	130	(21.1)
MBI-HSS depersonalization		
Mean (SD)	2.7	(3.3)
Low	517	(83.7)
Moderate	84	(13.6)
High	17	(2.8)
MBI-HSS personal accomplishment		
Mean (SD)	27.2	(9.6)
Low	56	(9.4)
Moderate	111	(18.7)
High	428	(71.9)
FATCOD-B short-form I score		
Mean (SD)	10.0	(2.0)
FATCOD-B short-form II score		
Mean (SD)	11.6	(1.9)

SD, standard deviation; PHQ-9, Patient Health Questionnaire-9; MBI-HSS, Maslach Burnout Inventory Human Services Survey; FATCOD-B, Frommelt Attitude Toward Care of Dying Scale—Form B.

### Psychological matters

The mean scores of EE, DP, and PA were 19.7 (11.2), 2.7 (3.3), and 27.2 (9.6), respectively (Table 1). Participants had a severe level of burnout at EE, DP, and PA of 21.1%, 2.8%, and 71.9%, respectively. The mean score of K-6 was 3.7 (4.0), and 4.1% of participants had suspected serious mental distress. The mean score of PHQ-9 was 5.6 (4.4), and 17.9% of participants were considered to have depression. The mean scores of FATCOD I and FATCOD II were 10.0 (2.0) and 11.6 (1.9), respectively.

### Experiences, approaches, and perspectives during nutritional counseling

**FIG. 2.**
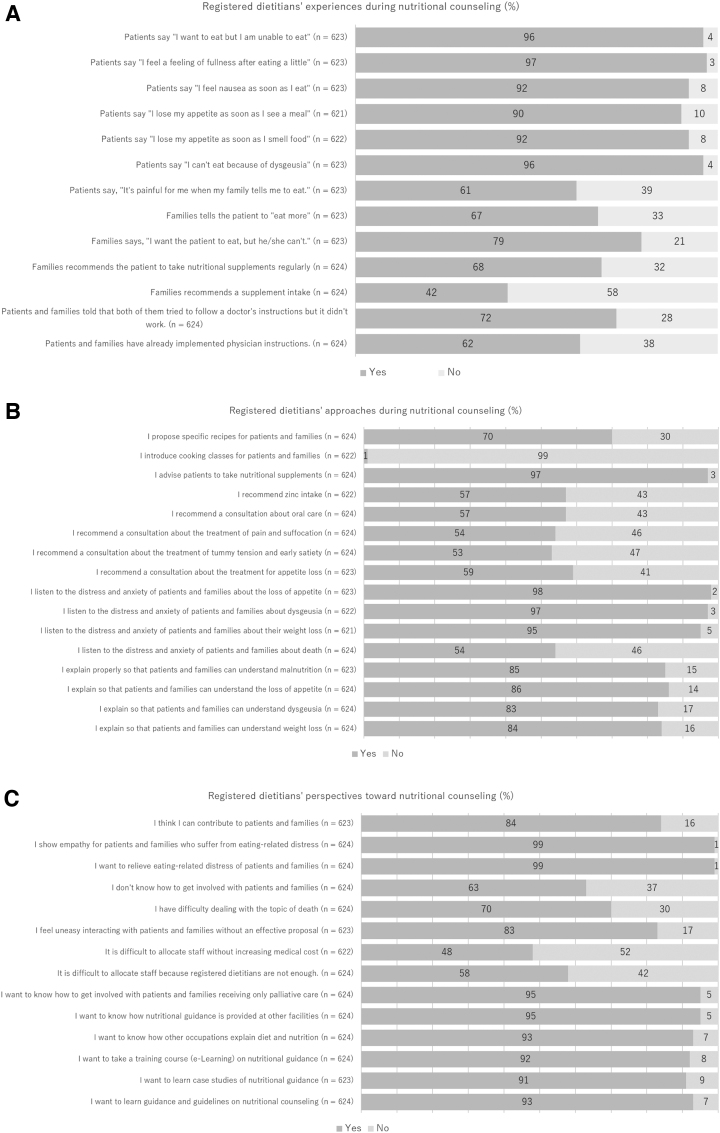
Registered dietitians' experiences, approaches, and perspectives during nutritional counseling. **(A)** Registered dietitians' experiences during nutritional counseling. **(B)** Registered dietitians' approaches during nutritional counseling. **(C)** Registered dietitians' perspectives during nutritional counseling.

More than 90% of participants experienced patients' suffering (Fig. 2A). However, the proportion of participants who experienced families' suffering was low compared with those of patients. More than 90% of participants recommended a nutritional supplement and listened to the distress and anxiety of patients and families (Fig. 2B). On the contrary, fewer participants recommended a consultation about treatment of symptoms or listened to the patient's distress and anxiety about death. Almost all of the dietitians wanted to relieve their patients' distress; however, they felt uneasy about getting involved with the family without an effective proposal or to dealing with the topic of death (Fig. 2C).

### Independent factors for burnout among registered dietitians

The results of univariate analyses for EE, DP, and PA are shown in Supplementary Tables S1–S3, respectively. The multivariate analyses for EE, DP, and PA are shown in Tables 2–4, respectively. A higher score on the EE was significantly associated with the approach of listening to the patients' and families' distress and anxiety about death (*p* = 0.034), the uneasiness interacting with patients and families without an effective proposal (*p* = 0.043), the difficulty of allocating staff without increasing medical cost (*p* < 0.001), more overtime hours (*p* < 0.001), a higher score of PHQ-9 (*p* < 0.001), and a higher score of K-6 (*p* < 0.001) (Table 2).

**Table 2.** Multivariate Linear Regression Analysis for High Score on Emotional Exhaustion

	***B***	**(95% CI)**	**SE**	***β***	***p***
Overtime hours per month	0.111	(0.069 − 0.154)	0.022	0.147	<0.0001^*^
PHQ-9 score	1.13	(0.901 − 1.359)	0.116	0.446	<0.0001^*^
K-6 score	0.793	(0.542 − 1.045)	0.128	0.282	<0.0001^*^
FATCOD-B short-form I score	−0.249	(−0.603 − 0.105)	0.18	−0.044	0.167
Patients and families told that both of them tried to follow a doctor's instructions but it didn't work	−0.133	(−0.853 − 0.588)	0.367	−0.011	0.718
Patients and families have already implemented physician instructions	0.218	(−0.451 − 0.886)	0.34	0.019	0.522
I listen to the distress and anxiety of patients and families about their weight loss	0.094	(−1.468 − 1.656)	0.795	0.003	0.906
I listen to the distress and anxiety of the patients and families about death	0.691	(0.053 − 1.329)	0.325	0.061	0.034^*^
I think I can contribute to patients and families	−0.162	(−1.039 − 0.715)	0.446	−0.01	0.717
I don't know how to get involved with patients and families	0.035	(−0.748 − 0.817)	0.398	0.003	0.931
I have difficulty dealing with the topic of death	−0.672	(−1.52 − 0.177)	0.432	−0.054	0.121
I feel uneasy interacting with patients and families without an effective proposal	1.026	(0.032 − 2.02)	0.506	0.069	0.043^*^
It is difficult to allocate staff without increasing medical cost	1.51	(0.705 − 2.314)	0.41	0.134	<0.001^*^
It is difficult to allocate staff because registered dietitians are not enough	0.03	(−0.775 − 0.835)	0.41	0.003	0.941
I want to know how to get involved with patients and families receiving only palliative care	0.007	(−1.562 − 1.575)	0.798	0	0.994
I want to know how other occupations explain diet and nutrition	0.44	(−0.925 − 1.805)	0.695	0.019	0.527
I want to learn case studies of nutritional guidance	−0.127	(−1.553 − 1.299)	0.726	−0.007	0.861
I want to learn guidance and guidelines on nutritional counseling	0.617	(−1.027 − 2.261)	0.837	0.026	0.461

^*^ *p* < 0.05.

*B*, partial regression coefficient; SE, standard error; β, standard partial regression coefficient.

**Table 3.** Multivariate Linear Regression Analysis for High Score on Depersonalization

	***B***	**(95% CI)**	**SE**	***β***	***p***
Years of clinical experience	−0.036	(−0.064 to −0.008)	0.014	−0.105	0.012^*^
Overtime hours per month	0.022	(0.004 to 0.04)	0.009	0.095	0.019^*^
PHQ-9 score	0.054	(−0.042 to 0.149)	0.049	0.071	0.269
K-6 score	0.259	(0.153 to 0.364)	0.054	0.305	<0.0001^*^
FATCOD-B short-form I score	−0.121	(−0.267 to 0.026)	0.074	−0.072	0.106
FATCOD-B short-form II score	0.139	(−0.001 to 0.279)	0.071	0.076	0.051
Patients and families told that both of them tried to follow a doctor's instructions but it didn't work	0.028	(−0.274 to 0.33)	0.154	0.007	0.856
Patients and families have already implemented physician instructions	−0.017	(−0.296 to 0.262)	0.142	−0.005	0.905
I propose specific recipes for patients and families	−0.356	(−0.654 to −0.057)	0.152	−0.096	0.020^*^
I recommend a consultation about oral care	−0.143	(−0.418 to 0.131)	0.14	−0.042	0.306
I listen to the distress and anxiety of patients and families about their weight loss	0.502	(−0.159 to 1.163)	0.337	0.059	0.137
I think I can contribute to patients and families	−0.041	(−0.41 to 0.327)	0.188	−0.009	0.825
I don't know how to get involved with patients and families	0.283	(−0.041 to 0.606)	0.165	0.08	0.087
I have difficulty dealing with the topic of death	−0.009	(−0.362 to 0.344)	0.18	−0.002	0.961
I feel uneasy interacting with patients and families without an effective proposal	0.086	(−0.325 to 0.498)	0.209	0.019	0.681
It is difficult to allocate staff without increasing medical cost	0.062	(−0.276 to 0.4)	0.172	0.018	0.718
It is difficult to allocate staff because registered dietitians are not enough.	0.083	(−0.255 to 0.422)	0.172	0.024	0.629
I want to know how nutritional guidance is provided at other facilities	−0.568	(−1.23 to 0.095)	0.337	−0.073	0.093
I want to learn case studies of nutritional guidance	−0.548	(−1.128 to 0.033)	0.296	−0.094	0.065
I want to learn guidance and guidelines on nutritional counseling	0.909	(0.225 to 1.592)	0.348	0.129	0.009^*^

^*^ *p* < 0.05.

**Table 4.** Multivariate Linear Regression Analysis for Low Score on Personal Accomplishment

	***B***	**(95% CI)**	**SE**	***β***	***p***
PHQ-9 score	0.153	(−0.109 to 0.415)	0.133	0.07	0.251
K-6 score	0.306	(0.018 to 0.595)	0.147	0.126	0.038^*^
FATCOD-B short-form I score	−0.783	(−1.199 to −0.367)	0.212	−0.163	<0.001^*^
FATCOD-B short-form II score	−1.054	(−1.441 to −0.666)	0.197	−0.202	<0.001^*^
Patients say “I feel a feeling of fullness after eating a little”	−0.764	(−2.843 to 1.314)	1.058	−0.028	0.47
Patients say “I lose my appetite as soon as I see a meal”	−1.362	(−2.699 to −0.024)	0.681	−0.087	0.046^*^
Patients say “I lose my appetite as soon as I smell food”	−0.103	(−1.579 to 1.373)	0.751	−0.006	0.891
Patients say, “It's painful for me when my family tells me to eat”	0.06	(−0.851 to 0.971)	0.464	0.006	0.897
Family tells the patient to “eat more”	−0.16	(−1.08 to 0.759)	0.468	−0.016	0.732
Family recommends the patient takes nutritional supplements regularly	−0.55	(−1.397 to 0.297)	0.431	−0.053	0.203
Family recommends supplement intake	0.05	(−0.773 to 0.874)	0.419	0.005	0.904
I propose specific recipes for patients and families	0.085	(−0.755 to 0.924)	0.427	0.008	0.843
I recommend zinc intake	−0.687	(−1.437 to 0.064)	0.382	−0.071	0.073
I recommend a consultation about oral care	−0.039	(−0.877 to 0.798)	0.426	−0.004	0.927
I recommend a consultation about the treatment of pain and suffocation	0.13	(−0.801 to 1.062)	0.474	0.013	0.783
I recommend a consultation about the treatment of tummy tension and early satiety	−0.217	(−1.16 to 0.725)	0.48	−0.022	0.651
I recommend a consultation about the treatment for appetite loss	−0.34	(−1.193 to 0.512)	0.434	−0.035	0.433
I listen to the distress and anxiety of patients and families about the loss of appetite	0.088	(−3.048 to 3.225)	1.597	0.002	0.956
I listen to the distress and anxiety of patients and families about dysgeusia	−1.127	(−3.931 to 1.676)	1.427	−0.04	0.43
I listen to the distress and anxiety of patients and families about their weight loss	−1.067	(−3.162 to 1.028)	1.067	−0.045	0.318
I listen to the patients' and families' distress and anxiety about death	−0.358	(−1.13 to 0.413)	0.393	−0.037	0.362
I explain properly so that patients and families can understand malnutrition	−1.674	(−3.084 to −0.264)	0.718	−0.124	0.020^*^
I explain so that patients and families can understand the loss of appetite	0.784	(−0.943 to 2.512)	0.88	0.057	0.373
I explain so that patients and families can understand dysgeusia	−0.551	(−2.082 to 0.981)	0.78	−0.042	0.48
I explain so that patients and families can understand weight loss	1.074	(−0.363 to 2.512)	0.732	0.082	0.143
I think I can contribute to patients and families	−1.514	(−2.596 to −0.433)	0.551	−0.115	0.006^*^
I show empathy for patients and families who suffer from eating-related distress	−1.057	(−4.731 to 2.616)	1.87	−0.024	0.572
I want to relieve eating-related distress of patients and families	0.913	(−4.351 to 6.176)	2.679	0.014	0.734
I don't know how to get involved with patients and families	0.814	(−0.088 to 1.715)	0.459	0.081	0.077
I have difficulty dealing with the topic of death	0.976	(−0.022 to 1.974)	0.508	0.092	0.055
I feel uneasy interacting with patients and families without an effective proposal	0.006	(−1.128 to 1.14)	0.577	0.0004	0.992
I want to know how other occupations explain diet and nutrition	0.307	(−1.304 to 1.917)	0.82	0.015	0.709
I want to learn case studies of nutritional guidance	0.082	(−1.265 to 1.429)	0.686	0.005	0.905

^*^ *p* < 0.05.

A higher score on DP was significantly associated with the absence of experience with the proposal of specific recipes for patients and families (*p* = 0.020), the need of guidance and guidelines on nutritional counseling (*p* = 0.009), fewer years of clinical experience (*p* = 0.012), more overtime hours (*p* = 0.019), and a higher score of K-6 (*p* < 0.001) (Table 3). A lower score on PA was significantly associated with the absence of experience of patients' appetite loss soon after they see a meal (*p* = 0.046), the absence of a proper explanation about malnutrition (*p* = 0.020), the absence of feeling of a worthwhile contribution to patients and families (*p* = 0.006), a lower score of FATCOD I (*p* < 0.001), a lower score of FATCOD II (*p* < 0.001), and a higher score of K-6 (*p* = 0.038) (Table 4).

## Discussion

To the best of our knowledge, the present study is the first to assess the experiences and perspectives during nutrition counseling, the prevalence of burnout, and burnout-associated factors among registered dietitians. The most important findings were that burnout on PA was quite high among registered dietitians for cancer patients, compared with other oncology staff.^11,17–19^ On the contrary, the proportion of those experiencing burnout on EE was similar, and the proportion of those on DP was low compared with other oncology staff.^11,17–19^ Years of clinical experience, overtime hours, and severe stress were similar to other oncology staff.^11,17–19^

Furthermore, the present study revealed that burnout-associated factors among registered dietitians were experiences during nutrition counseling, the feeling of contribution, and not so positive attitude for caring dying patients. If registered dietitians encounter patients feeling appetite loss soon after they see a meal or they try to properly explain about malnutrition, burnout on PA may improve. Registered dietitians continuously encounter cancer patients and families with severe eating-related distress in daily practice. Moreover, appetite loss and malnutrition among advanced cancer patients are refractory to nutritional intervention. Thus, registered dietitians experienced the deterioration of patients' physical condition and their dying. Such an illness trajectory among cancer patients may evoke severe stress, compassion fatigue, and decreased professional QOL for registered dietitians such as hospice nurses and social workers.^7,8^

A previous study that drew attention to the stress and burnout among registered dietitians recommended close communication and collaboration between health care professionals and the education or training of resilience, mindfulness, and empathy to prevent stress and burnout.^19,25^ For other oncology professionals, training improved their burnout.^26–30^ Our findings suggest that most registered dietitians felt anxiety toward cancer patients, and almost all of them wanted to learn better nutrition counseling and increase their skills to provide better services. A trial for integrating counseling psychology into nutrition practice had been proposed.^31^ Therefore, the development or modification of the education system for nutritional intervention, including psychological support and self-management toward registered dietitians on oncology fields or toward students in the faculty of nutrition, will improve their stress, prevent burnout, and assist them in providing better care for cancer patients and families.

The present study was a nationwide study to reveal the experiences, approaches, and perspectives among registered dietitians. Most of the registered dietitians experienced eating-related problems from the patients themselves. Although a previous study showed that cancer patients felt distress originating from their relationship with their families,^32^ only 60% of the registered dietitians reported hearing about patients' stress when their family told them to eat. If registered dietitians spend more time with individual patients and learn psychological counseling skills, they will be able to listen to patients more effectively. On the approaches during nutrition counseling, only 60% of registered dietitians recommended a consultation about nutritional impact symptoms including pain, oral care, early satiety and appetite loss. Nutritional impact symptoms are associated with malnutrition,^33,34^ and thus, screening of treatable nutritional impact symptoms and consultation with other professionals are recommended to improve patients' symptom burdens and to increase their QOL.

The present study had several limitations. First, because the present study did not enroll all registered dietitians who belonged to each designated cancer hospital, our findings may not be generalizable. Second, the definition of “moderately experienced registered dietitian” and “inexperienced registered dietitian” was unclear. The composition of years of experiences for registered dietitians varies from each institute. Third, the present study was conducted in Japan. Our findings may not be generalizable for other countries. However, the burnout among oncologists and palliative care physicians is a common problem in the world. Therefore, the burnout among registered dietitians may also be a common problem. Forth, there may be a response bias because of the cross-sectional design. Among the invited registered dietitians, registered dietitians with severe distress tended to answer the present questionnaires. Fifth, marital state was not investigated. This is a correlated factor for burnout. Therefore, present findings might be changeable by marital state.

Sixth, the present survey might be affected by pandemic-related restrictions due to COVID-19. This might affect the prevalence of burnout. Seventh, there was a multiple testing problem. However, this is the first exploratory study to investigate burnout-associated factors among registered dietitians in oncology fields. Registered dietitians play an important role in managing nutrition care among cancer patients.

## Conclusion

The present study revealed the experiences and perspectives during nutrition counseling, the prevalence of burnout, and burnout-associated factors among registered dietitians in oncology fields. The prevalence of burnout on PA was quite high. Therefore, registered dietitians who engaged in nutritional counseling for cancer patients and families might benefit from education to help protect against burnout.

## Supplementary Material

Supplemental data

## Abbreviations Used

DP: depersonalization
EE: emotional exhaustion
FATCOD: Frommelt Attitude Toward Care of the Dying
FATCOD-B: Frommelt Attitude Toward Care of Dying Scale - Form B
MBI-HSS: Maslach Burnout Inventory Human Services Survey
PA: personal accomplishment
PHQ-9: Patient Health Questionnaire-9
QOL: quality of life
SD: standard deviation
SE: standard error
