# Supplementary material for: A Japanese Nationwide Survey of Nutritional Counseling for Cancer Patients and Risk Factors of Burnout among Registered Dietitians
Source: Palliat Med Rep. 2022 Sep 27;3(1):211–9. doi: 10.1089/pmr.2022.0038 (PMC9983137; doi:10.1089/pmr.2022.0038)
Supplement: Supplemental data [file Suppl_TableS1-S3.docx]

Supplemental Table 1. Univariate analysis of emotional exhaustion

|  |  |  | N | Mean | SD | p-value |
| --- | --- | --- | --- | --- | --- | --- |
| Backgrounds | |  |  |  |  |  |
|  | Sex | Male | 62 | 18.1 | 12.4 | 0.159 |
|  |  | Female | 546 | 19.8 | 11.1 |  |
|  | Years of clinical experience | B, SE |  | -0.014 | 0.046 | 0.77 |
|  | Number of beds | < 200 | 8 | 24.1 | 12.8 | 0.735 |
|  |  | 201 - 400 | 128 | 19 | 11.1 |  |
|  |  | 401 - 600 | 248 | 20 | 11.4 |  |
|  |  | 601 - 1000 | 193 | 19.6 | 11.3 |  |
|  |  | > 1000 | 29 | 18.8 | 9.7 |  |
|  | Number of registered dietitians | < 3 | 24 | 18.9 | 10 | 0.954 |
|  |  | 4 - 6 | 169 | 19.6 | 11.3 |  |
|  |  | 7 - 10 | 263 | 20.1 | 11.5 |  |
|  |  | > 10 | 151 | 19.2 | 10.7 |  |
|  | Overtime hours per month | B, SE |  | 0.16 | 0.031 | <.0001 |
| Psychological matters | |  |  |  |  |  |
|  | PHQ-9 score | B, SE |  | 1.776 | 0.073 | <.0001 |
|  | K-6 score | B, SE |  | 1.873 | 0.085 | <.0001 |
|  | FATCOD-B short-form I score | B, SE |  | -1.045 | 0.217 | <.0001 |
|  | FATCOD-B short-form II score | B, SE |  | 0.169 | 0.241 | 0.484 |
| Experiences | |  |  |  |  |  |
|  | Patients say "I want to eat but I am unable to eat" | No | 22 | 16.7 | 8.7 | 0.274 |
|  |  | Yes | 587 | 19.8 | 11.3 |  |
|  | Patients say "I feel a feeling of fullness after eating a little" | No | 20 | 19.7 | 11.6 | 0.774 |
|  |  | Yes | 589 | 19.7 | 11.2 |  |
|  | Patients say "I feel nausea as soon as I eat" | No | 47 | 19.4 | 10.8 | 0.99 |
|  |  | Yes | 562 | 19.7 | 11.2 |  |
|  | Patients say "I lose my appetite as soon as I see a meal" | No | 62 | 17.3 | 11.2 | 0.087 |
|  |  | Yes | 545 | 20 | 11.1 |  |
|  | Patients say "I lose my appetite as soon as I smell food" | No | 52 | 18.4 | 11 | 0.412 |
|  |  | Yes | 556 | 19.8 | 11.2 |  |
|  | Patients say "I can't eat because of dysgeusia" | No | 26 | 20.6 | 11.1 | 0.518 |
|  |  | Yes | 583 | 19.7 | 11.2 |  |
|  | Patients say, "It's painful for me when my family tells me to eat." | No | 236 | 19.2 | 11 | 0.449 |
|  |  | Yes | 374 | 20 | 11.3 |  |
|  | Families tells the patient to "eat more" | No | 199 | 18.6 | 11 | 0.107 |
|  |  | Yes | 411 | 20.2 | 11.3 |  |
|  | Families says, "I want the patient to eat, but he/she can't." | No | 126 | 19.5 | 10.7 | 0.815 |
|  |  | Yes | 483 | 19.7 | 11.3 |  |
|  | Families recommends the patient to take nutritional supplements regularly | No | 197 | 19.9 | 10.8 | 0.556 |
|  |  | Yes | 413 | 19.6 | 11.4 |  |
|  | Families recommends a supplement intake | No | 351 | 19.3 | 11.3 | 0.266 |
|  |  | Yes | 259 | 20.2 | 11.1 |  |
|  | Patients and families told that both of them tried to follow a doctor's instructions but it didn't work. | No | 172 | 18.4 | 11 | 0.097 |
|  |  | Yes | 438 | 20.2 | 11.2 |  |
|  | Patients and families have already implemented physician instructions. | No | 231 | 18.7 | 11.5 | 0.052 |
|  |  | Yes | 379 | 20.3 | 10.9 |  |
| Approaches | |  |  |  |  |  |
|  | I propose specific recipes for patients and families | No | 186 | 19.8 | 11.6 | 0.964 |
|  |  | Yes | 424 | 19.6 | 11 |  |
|  | I introduce cooking classes for patients and families | No | 602 | 19.8 | 11.1 | 0.21 |
|  |  | Yes | 6 | 15 | 15.2 |  |
|  | I advise patients to take nutritional supplements | No | 20 | 19.4 | 13.3 | 0.758 |
|  |  | Yes | 590 | 19.7 | 11.1 |  |
|  | I recommend zinc intake | No | 260 | 19.9 | 11.6 | 0.95 |
|  |  | Yes | 348 | 19.6 | 10.9 |  |
|  | I recommend a consultation about oral care | No | 262 | 20.1 | 11.2 | 0.314 |
|  |  | Yes | 348 | 19.4 | 11.2 |  |
|  | I recommend a consultation about the treatment of pain and suffocation | No | 279 | 19.4 | 11 | 0.588 |
|  |  | Yes | 331 | 20 | 11.3 |  |
|  | I recommend a consultation about the treatment of tummy tension and early satiety | No | 289 | 19.4 | 11.1 | 0.574 |
|  |  | Yes | 321 | 19.9 | 11.2 |  |
|  | I recommend a consultation about the treatment for appetite loss | No | 247 | 18.9 | 10.9 | 0.125 |
|  |  | Yes | 362 | 20.3 | 11.3 |  |
|  | I listen to the distress and anxiety of patients and families about the loss of appetite | No | 12 | 21.2 | 16.8 | 0.885 |
|  |  | Yes | 597 | 19.7 | 11.1 |  |
|  | I listen to the distress and anxiety of patients and families about dysgeusia | No | 18 | 17.7 | 12.6 | 0.487 |
|  |  | Yes | 590 | 19.8 | 11.1 |  |
|  | I listen to the distress and anxiety of patients and families about their weight loss | No | 27 | 15.8 | 10.5 | 0.083 |
|  |  | Yes | 580 | 19.9 | 11.2 |  |
|  | I listen to the distress and anxiety of patients and families about death | No | 277 | 18.6 | 11.4 | 0.023 |
|  |  | Yes | 333 | 20.6 | 10.9 |  |
|  | I explain properly so that patients and families can understand malnutrition | No | 87 | 19.9 | 10.5 | 0.735 |
|  |  | Yes | 522 | 19.6 | 11.3 |  |
|  | I explain so that patients and families can understand the loss of appetite | No | 84 | 21 | 11.1 | 0.178 |
|  |  | Yes | 526 | 19.5 | 11.2 |  |
|  | I explain so that patients and families can understand dysgeusia | No | 99 | 21.1 | 11.4 | 0.174 |
|  |  | Yes | 511 | 19.4 | 11.1 |  |
|  | I explain so that patients and families can understand weight loss | No | 93 | 20.2 | 10.7 | 0.452 |
|  |  | Yes | 517 | 19.6 | 11.3 |  |
| Perspectives | |  |  |  |  |  |
|  | I think I can contribute to patients and families | No | 97 | 22.9 | 11.4 | 0.002 |
|  |  | Yes | 514 | 19.1 | 11.1 |  |
|  | I show empathy for patients and families who suffer from eating-related distress | No | 9 | 20.9 | 16.1 | 0.94 |
|  |  | Yes | 603 | 19.7 | 11.1 |  |
|  | I want to relieve eating-related distress of patients and families | No | 3 | 13 | 8.5 | 0.287 |
|  |  | Yes | 609 | 19.7 | 11.2 |  |
|  | I don't know how to get involved with patients and families | No | 222 | 17 | 10.7 | <.0001 |
|  |  | Yes | 390 | 21.2 | 11.2 |  |
|  | I have difficulty dealing with the topic of death | No | 181 | 17.2 | 10.6 | 0.003 |
|  |  | Yes | 431 | 20.7 | 11.2 |  |
|  | I feel uneasy interacting with patients and families without an effective proposal | No | 107 | 14.3 | 9.2 | <.0001 |
|  |  | Yes | 504 | 20.8 | 11.2 |  |
|  | It is difficult to allocate staff without increasing medical cost | No | 318 | 17.5 | 10.5 | <.0001 |
|  |  | Yes | 292 | 22 | 11.5 |  |
|  | It is difficult to allocate staff because registered dietitians are not enough. | No | 258 | 17.4 | 10.5 | <.0001 |
|  |  | Yes | 353 | 21.3 | 11.4 |  |
|  | I want to know how to get involved with patients and families receiving only palliative care | No | 29 | 14.3 | 10 | 0.008 |
|  |  | Yes | 583 | 19.9 | 11.2 |  |
|  | I want to know how nutritional guidance is provided at other facilities | No | 31 | 17.3 | 13.5 | 0.108 |
|  |  | Yes | 581 | 19.8 | 11 |  |
|  | I want to know how other occupations explain diet and nutrition | No | 41 | 15.4 | 9.6 | 0.012 |
|  |  | Yes | 571 | 20 | 11.2 |  |
|  | I want to take a training course (e-Learning) on ​​nutritional guidance | No | 51 | 18 | 11.6 | 0.172 |
|  |  | Yes | 561 | 19.8 | 11.1 |  |
|  | I want to learn case studies of nutritional guidance | No | 57 | 16.5 | 12 | 0.011 |
|  |  | Yes | 554 | 20 | 11 |  |
|  | I want to learn guidance and guidelines on nutritional counseling | No | 42 | 16.5 | 11.6 | 0.03 |
|  |  | Yes | 570 | 19.9 | 11.1 |  |
| SD, standard deviation; B, partial regression coefficient; SE, standard error; PHQ-9, Patient Health Questionnaire-9; FATCOD-B, Frommelt Attitude Toward Care of Dying Scale - Form B | | | | | | |

Supplemental Table 2. Univariate analysis of depersonalization

|  |  |  | N | Mean | SD | p-value |
| --- | --- | --- | --- | --- | --- | --- |
| Backgrounds |  |  |  |  |  |  |
|  | Sex | Male | 64 | 2.6 | 3.3 | 0.75 |
|  |  | Female | 547 | 2.7 | 3.4 |  |
|  | Years of clinical experience | B, SE |  | -0.042 | 0.014 | 0.003 |
|  | Number of beds | < 200 | 8 | 4.3 | 4.6 | 0.635 |
|  |  | 201 - 400 | 129 | 2.8 | 3.4 |  |
|  |  | 401 - 600 | 252 | 2.7 | 3.4 |  |
|  |  | 601 - 1000 | 191 | 2.5 | 3.2 |  |
|  |  | > 1000 | 29 | 2.2 | 3.3 |  |
|  | Number of registered dietitians | < 3 | 24 | 1.6 | 1.6 | 0.188 |
|  |  | 4 - 6 | 171 | 2.8 | 3.7 |  |
|  |  | 7 - 10 | 264 | 2.9 | 3.4 |  |
|  |  | > 10 | 151 | 2.3 | 3 |  |
|  | Overtime hours per month | B, SE |  | 0.024 | 0.009 | 0.009 |
| Psychological matters |  |  |  |  |  |  |
|  | PHQ-9 score | B, SE |  | 0.274 | 0.029 | <.0001 |
|  | K-6 score | B, SE |  | 0.332 | 0.031 | <.0001 |
|  | FATCOD-B short-form I score | B, SE |  | -0.302 | 0.065 | <.0001 |
|  | FATCOD-B short-form II score | B, SE |  | 0.118 | 0.072 | 0.102 |
| Experiences |  |  |  |  |  |  |
|  | Patients say "I want to eat but I am unable to eat" | No | 21 | 3.1 | 3.1 | 0.347 |
|  |  | Yes | 591 | 2.7 | 3.4 |  |
|  | Patients say "I feel a feeling of fullness after eating a little" | No | 20 | 1.9 | 2.1 | 0.496 |
|  |  | Yes | 592 | 2.7 | 3.4 |  |
|  | Patients say "I feel nausea as soon as I eat" | No | 47 | 2.4 | 2.7 | 0.78 |
|  |  | Yes | 565 | 2.7 | 3.4 |  |
|  | Patients say "I lose my appetite as soon as I see a meal" | No | 62 | 2.1 | 2.7 | 0.15 |
|  |  | Yes | 548 | 2.7 | 3.4 |  |
|  | Patients say "I lose my appetite as soon as I smell food" | No | 52 | 2.5 | 3.1 | 0.751 |
|  |  | Yes | 559 | 2.7 | 3.4 |  |
|  | Patients say "I can't eat because of dysgeusia" | No | 26 | 2.2 | 2.9 | 0.456 |
|  |  | Yes | 586 | 2.7 | 3.4 |  |
|  | Patients say, "It's painful for me when my family tells me to eat." | No | 236 | 2.7 | 3.4 | 0.863 |
|  |  | Yes | 376 | 2.6 | 3.3 |  |
|  | Family tells the patient to "eat more" | No | 200 | 2.5 | 3.3 | 0.254 |
|  |  | Yes | 412 | 2.8 | 3.4 |  |
|  | Family says, "I want the patient to eat, but he/she can't." | No | 126 | 2.8 | 3.7 | 0.835 |
|  |  | Yes | 486 | 2.6 | 3.3 |  |
|  | Families recommends the patient takes nutritional supplements regularly | No | 197 | 2.6 | 3.3 | 0.384 |
|  |  | Yes | 416 | 2.7 | 3.4 |  |
|  | Families recommends supplement intake | No | 354 | 2.7 | 3.4 | 0.78 |
|  |  | Yes | 259 | 2.7 | 3.2 |  |
|  | Patients and families told that both of them tried to follow a doctor's instructions but it didn't work. | No | 172 | 2.4 | 3.6 | 0.015 |
|  |  | Yes | 441 | 2.8 | 3.3 |  |
|  | Patients and families have already implemented physician instructions. | No | 234 | 2.4 | 3.3 | 0.084 |
|  |  | Yes | 379 | 2.8 | 3.4 |  |
| Approaches |  |  |  |  |  |  |
|  | I propose specific recipes for patients and families | No | 187 | 3.2 | 3.8 | 0.012 |
|  |  | Yes | 426 | 2.4 | 3.1 |  |
|  | I introduce cooking classes for patients and families | No | 606 | 2.7 | 3.3 | 0.729 |
|  |  | Yes | 5 | 4.6 | 5.9 |  |
|  | I advise patients to take nutritional supplements | No | 20 | 4.4 | 4.5 | 0.106 |
|  |  | Yes | 593 | 2.6 | 3.3 |  |
|  | I recommend zinc intake | No | 263 | 2.9 | 3.8 | 0.44 |
|  |  | Yes | 348 | 2.5 | 3 |  |
|  | I recommend a consultation about oral care | No | 263 | 3 | 3.5 | 0.02 |
|  |  | Yes | 350 | 2.4 | 3.3 |  |
|  | I recommend a consultation about the treatment of pain and suffocation | No | 279 | 2.6 | 3.4 | 0.641 |
|  |  | Yes | 334 | 2.7 | 3.4 |  |
|  | I recommend a consultation about the treatment of tummy tension and early satiety | No | 289 | 2.6 | 3.3 | 0.494 |
|  |  | Yes | 324 | 2.7 | 3.4 |  |
|  | I recommend a consultation about treatment for appetite loss | No | 248 | 2.8 | 3.3 | 0.175 |
|  |  | Yes | 364 | 2.6 | 3.4 |  |
|  | I listen to the distress and anxiety of patients and families about the loss of appetite | No | 12 | 3.8 | 4.5 | 0.52 |
|  |  | Yes | 600 | 2.6 | 3.3 |  |
|  | I listen to the distress and anxiety of patients and families about dysgeusia | No | 18 | 2.8 | 4.2 | 0.695 |
|  |  | Yes | 593 | 2.7 | 3.3 |  |
|  | I listen to the distress and anxiety of patients and families about their weight loss | No | 27 | 1.9 | 3.5 | 0.067 |
|  |  | Yes | 583 | 2.7 | 3.3 |  |
|  | I listen to the distress and anxiety of patients and families about death | No | 281 | 2.7 | 3.2 | 0.718 |
|  |  | Yes | 332 | 2.7 | 3.4 |  |
|  | I explain properly so that patients and families can understand malnutrition | No | 88 | 3.1 | 3.6 | 0.186 |
|  |  | Yes | 524 | 2.6 | 3.3 |  |
|  | I explain so that patients and families can understand the loss of appetite | No | 85 | 3.1 | 3.7 | 0.249 |
|  |  | Yes | 528 | 2.6 | 3.3 |  |
|  | I explain so that patients and families can understand dysgeusia | No | 100 | 3.3 | 4.2 | 0.199 |
|  |  | Yes | 513 | 2.5 | 3.1 |  |
|  | I explain so that patients and families can understand weight loss | No | 94 | 2.9 | 3.6 | 0.74 |
|  |  | Yes | 519 | 2.6 | 3.3 |  |
| Perspectives |  |  |  |  |  |  |
|  | I think I can contribute to patients and families | No | 97 | 3.4 | 3.7 | 0.015 |
|  |  | Yes | 517 | 2.5 | 3.3 |  |
|  | I show empathy for patients and families who suffer from eating-related distress | No | 9 | 4.3 | 6.6 | 0.985 |
|  |  | Yes | 606 | 2.6 | 3.3 |  |
|  | I want to relieve eating-related distress of patients and families | No | 3 | 1.3 | 0.6 | 0.867 |
|  |  | Yes | 612 | 2.7 | 3.4 |  |
|  | I don't know how to get involved with patients and families | No | 224 | 1.8 | 2.8 | <.0001 |
|  |  | Yes | 391 | 3.1 | 3.5 |  |
|  | I have difficulty dealing with the topic of death | No | 182 | 2 | 3 | <.0001 |
|  |  | Yes | 433 | 3 | 3.4 |  |
|  | I feel uneasy interacting with patients and families without an effective proposal | No | 106 | 1.4 | 2.4 | <.0001 |
|  |  | Yes | 508 | 2.9 | 3.5 |  |
|  | It is difficult to allocate staff without increasing medical cost | No | 320 | 2.3 | 3.2 | 0.004 |
|  |  | Yes | 293 | 3 | 3.5 |  |
|  | It is difficult to allocate staff because registered dietitians are not enough. | No | 260 | 2.3 | 3.1 | 0.003 |
|  |  | Yes | 354 | 2.9 | 3.5 |  |
|  | I want to know how to get involved with patients and families receiving only palliative care | No | 29 | 2.5 | 3.8 | 0.521 |
|  |  | Yes | 586 | 2.7 | 3.3 |  |
|  | I want to know how nutritional guidance is provided at other facilities | No | 31 | 2.6 | 4.8 | 0.093 |
|  |  | Yes | 584 | 2.7 | 3.3 |  |
|  | I want to know how other occupations explain diet and nutrition | No | 41 | 2.1 | 2.8 | 0.273 |
|  |  | Yes | 574 | 2.7 | 3.4 |  |
|  | I want to take a training course (e-Learning) on ​​nutritional guidance | No | 51 | 2.3 | 3.5 | 0.391 |
|  |  | Yes | 564 | 2.7 | 3.3 |  |
|  | I want to learn case studies of nutritional guidance | No | 57 | 2.3 | 3.6 | 0.098 |
|  |  | Yes | 557 | 2.7 | 3.3 |  |
|  | I want to learn guidance and guidelines on nutritional counseling | No | 42 | 1.5 | 2.5 | 0.007 |
|  |  | Yes | 573 | 2.7 | 3.4 |  |
| SD, standard deviation; B, partial regression coefficient; SE, standard error; PHQ-9, Patient Health Questionnaire-9; FATCOD-B, Frommelt Attitude Toward Care of Dying Scale Form B | | | | | | |

Supplemental Table 3. Univariate analysis of personal accomplishment

|  |  |  | N | Mean | SD | p-value |
| --- | --- | --- | --- | --- | --- | --- |
| Backgrounds | |  |  |  |  |  |
|  | Sex | Male | 60 | 28.9 | 9.4 | 0.172 |
|  |  | Female | 528 | 27.1 | 9.6 |  |
|  | Years of clinical experience | B, SE |  | -0.018 | 0.041 | 0.655 |
|  | Number of beds | < 200 | 8 | 23.9 | 9.8 | 0.234 |
|  |  | 201 - 400 | 126 | 27.2 | 10.4 |  |
|  |  | 401 - 600 | 239 | 26.7 | 9 |  |
|  |  | 601 - 1000 | 185 | 28.3 | 9.7 |  |
|  |  | > 1000 | 28 | 25.9 | 10.1 |  |
|  | Number of registered dietitians | < 3 | 23 | 29.4 | 7.9 | 0.142 |
|  |  | 4 - 6 | 162 | 26 | 9.7 |  |
|  |  | 7 - 10 | 254 | 27.3 | 9.2 |  |
|  |  | > 10 | 148 | 28.4 | 10.2 |  |
|  | Overtime hours per month | B, SE |  | -0.032 | 0.027 | 0.251 |
| Pcyhological matters | |  |  |  |  |  |
|  | PHQ-9 score | B, SE |  | 0.564 | 0.086 | <.0001 |
|  | K-6 score | B, SE |  | 0.658 | 0.097 | <.0001 |
|  | FATCOD-B short-form I score | B, SE |  | -1.479 | 0.187 | <.0001 |
|  | FATCOD-B short-form II score | B, SE |  | -1.189 | 0.206 | <.0001 |
| Experiences | |  |  |  |  |  |
|  | Patients say "I want to eat but I am unable to eat" | No | 22 | 28.4 | 8.6 | 0.668 |
|  |  | Yes | 569 | 27.2 | 9.6 |  |
|  | Patients say "I feel a feeling of fullness after eating a little" | No | 20 | 23.8 | 9.2 | 0.072 |
|  |  | Yes | 570 | 27.4 | 9.6 |  |
|  | Patients say "I feel nausea as soon as I eat" | No | 47 | 26.2 | 9.3 | 0.488 |
|  |  | Yes | 543 | 27.4 | 9.6 |  |
|  | Patients say "I lose my appetite as soon as I see a meal" | No | 62 | 24 | 10.5 | 0.016 |
|  |  | Yes | 526 | 27.7 | 9.4 |  |
|  | Patients say "I lose my appetite as soon as I smell food" | No | 51 | 24.7 | 9.8 | 0.087 |
|  |  | Yes | 538 | 27.5 | 9.6 |  |
|  | Patients say "I can't eat because of dysgeusia" | No | 25 | 24.3 | 9.5 | 0.134 |
|  |  | Yes | 565 | 27.4 | 9.6 |  |
|  | Patients say, "It's painful for me when my family tells me to eat." | No | 230 | 26.4 | 9.2 | 0.058 |
|  |  | Yes | 360 | 27.8 | 9.8 |  |
|  | Families tells the patient to "eat more" | No | 194 | 26 | 9.5 | 0.031 |
|  |  | Yes | 396 | 27.9 | 9.6 |  |
|  | Families says, "I want the patient to eat, but he/she can't." | No | 122 | 26.5 | 9.8 | 0.459 |
|  |  | Yes | 468 | 27.5 | 9.6 |  |
|  | Families recommends the patient takes nutritional supplements regularly | No | 190 | 25.4 | 9.2 | 0.001 |
|  |  | Yes | 401 | 28.1 | 9.7 |  |
|  | Families recommends a supplement intake | No | 341 | 26.6 | 9.5 | 0.065 |
|  |  | Yes | 250 | 28.2 | 9.7 |  |
|  | Patients and families told that both of them tried to follow a doctor's instructions but it didn't work. | No | 163 | 28 | 9.9 | 0.176 |
|  |  | Yes | 428 | 27 | 9.5 |  |
|  | Patients and families have already implemented physician instructions. | No | 221 | 27.7 | 10.3 | 0.289 |
|  |  | Yes | 370 | 27 | 9.1 |  |
| Approaches | |  |  |  |  |  |
|  | I propose specific recipes for patients and families | No | 182 | 25.8 | 9.8 | 0.037 |
|  |  | Yes | 409 | 27.8 | 9.5 |  |
|  | I introduce cooking classes for patients and families | No | 584 | 27.2 | 9.6 | 0.273 |
|  |  | Yes | 5 | 22.2 | 9.9 |  |
|  | I advise patients to take nutritional supplements | No | 20 | 26.9 | 10.8 | 0.911 |
|  |  | Yes | 571 | 27.2 | 9.6 |  |
|  | I recommend zinc intake | No | 256 | 25.9 | 9.8 | 0.009 |
|  |  | Yes | 333 | 28.2 | 9.3 |  |
|  | I recommend a consultation about oral care | No | 254 | 25.7 | 9.6 | 0.003 |
|  |  | Yes | 337 | 28.3 | 9.5 |  |
|  | I recommend a consultation about the treatment of pain and suffocation | No | 269 | 25.9 | 9.9 | 0.006 |
|  |  | Yes | 322 | 28.3 | 9.2 |  |
|  | I recommend a consultation about the treatment of tummy tension and early satiety | No | 279 | 25.8 | 9.6 | 0.001 |
|  |  | Yes | 312 | 28.5 | 9.4 |  |
|  | I recommend a consultation about the treatment for appetite loss | No | 239 | 25.8 | 9.9 | 0.009 |
|  |  | Yes | 351 | 28.2 | 9.3 |  |
|  | I listen to the distress and anxiety of patients and families about the loss of appetite | No | 12 | 20.1 | 11.3 | 0.028 |
|  |  | Yes | 578 | 27.4 | 9.5 |  |
|  | I listen to the distress and anxiety of patients and families about dysgeusia | No | 18 | 20.4 | 10.9 | 0.009 |
|  |  | Yes | 571 | 27.4 | 9.5 |  |
|  | I listen to the distress and anxiety of patients and families about their weight loss | No | 27 | 22.9 | 9.8 | 0.027 |
|  |  | Yes | 561 | 27.4 | 9.6 |  |
|  | I listen to the patients and families distress and anxiety about death | No | 270 | 26 | 9.5 | 0.008 |
|  |  | Yes | 321 | 28.2 | 9.6 |  |
|  | I explain properly so that patients and families can understand malnutrition | No | 89 | 22.8 | 9.6 | <.0001 |
|  |  | Yes | 501 | 28 | 9.4 |  |
|  | I explain so that patients and families can understand the loss of appetite | No | 86 | 23.2 | 9.5 | <.0001 |
|  |  | Yes | 505 | 27.9 | 9.5 |  |
|  | I explain so that patients and families can understand dysgeusia | No | 98 | 23.1 | 9.5 | <.0001 |
|  |  | Yes | 493 | 28 | 9.4 |  |
|  | I explain so that patients and families can understand weight loss | No | 95 | 24 | 9.9 | 0.001 |
|  |  | Yes | 496 | 27.8 | 9.4 |  |
| Perspectives | |  |  |  |  |  |
|  | I think I can contribute to patients and families | No | 95 | 21.7 | 8.5 | <.0001 |
|  |  | Yes | 496 | 28.3 | 9.5 |  |
|  | I show empathy for patients and families who suffer from eating-related distress | No | 8 | 17 | 9.2 | 0.006 |
|  |  | Yes | 584 | 27.4 | 9.5 |  |
|  | I want to relieve eating-related distress of patients and families | No | 3 | 27.7 | 9.7 | 0.925 |
|  |  | Yes | 589 | 27.2 | 9.6 |  |
|  | I don't know how to get involved with patients and families | No | 214 | 30.4 | 9.3 | <.0001 |
|  |  | Yes | 378 | 25.5 | 9.3 |  |
|  | I have difficulty dealing with the topic of death | No | 174 | 30.8 | 9.5 | <.0001 |
|  |  | Yes | 418 | 25.8 | 9.3 |  |
|  | I feel uneasy interacting with patients and families without an effective proposal | No | 105 | 30.9 | 10.1 | <.0001 |
|  |  | Yes | 486 | 26.4 | 9.3 |  |
|  | It is difficult to allocate staff without increasing medical cost | No | 309 | 27 | 9.7 | 0.453 |
|  |  | Yes | 281 | 27.6 | 9.5 |  |
|  | It is difficult to allocate staff because registered dietitians are not enough. | No | 250 | 27.5 | 9.8 | 0.427 |
|  |  | Yes | 341 | 27 | 9.5 |  |
|  | I want to know how to get involved with patients and families receiving only palliative care | No | 28 | 28.1 | 9.4 | 0.639 |
|  |  | Yes | 564 | 27.2 | 9.6 |  |
|  | I want to know how nutritional guidance is provided at other facilities | No | 29 | 25.1 | 12 | 0.244 |
|  |  | Yes | 563 | 27.3 | 9.5 |  |
|  | I want to know how other occupations explain diet and nutrition | No | 38 | 29.6 | 10.7 | 0.079 |
|  |  | Yes | 554 | 27.1 | 9.5 |  |
|  | I want to take a training course (e-Learning) on ​​nutritional guidance | No | 45 | 28 | 10.6 | 0.581 |
|  |  | Yes | 547 | 27.2 | 9.5 |  |
|  | I want to learn case studies of nutritional guidance | No | 54 | 29.7 | 9.5 | 0.044 |
|  |  | Yes | 537 | 27 | 9.6 |  |
|  | I want to learn guidance and guidelines on nutritional counseling | No | 39 | 29.4 | 9.9 | 0.15 |
|  |  | Yes | 553 | 27.1 | 9.6 |  |
| SD, standard deviation; B, partial regression coefficient; SE, standard error; PHQ-9, Patient Health Questionnaire-9; FATCOD-B, Frommelt Attitude Toward Care of Dying Scale Form B | | | | | | |
